# The Laundry as Part of the Matron's Department

**Published:** 1909-09-25

**Authors:** 


### Nursing Administration.

### THE LAUNDRY AS PART OF THE MATRON'S DEPARTMENT.

Hospital authorities have perhaps hardly yet realised the extent to which modern surgical developments have magnified the *rôle* of clean linen in hospital treatment. Amidst all the elaborate precautions against dirt which have grown up round the patient's bed, it has been too often overlooked that some of the most important are those relating to the bed itself; that it matters intensely through what cleansing process the sheets have lately passed; that "washing" may be only another name for infecting, and that the purifying of linen for the patient's use is far too serious a detail in his cure to be entrusted to ignorant people under no inspection, acting on purely commercial lines. Since the issue of separate washing returns at the instance of the King's Fund last year, it has been possible to compare the cost of washing done in outside establishments and the cost of the hospital private laundry. From the tables of comparison thus afforded, it has become evident that there is very little to choose in the matter of expense between the two methods of administration. The cost of the outside laundry varies between £10 10s. per bed at the Miller Hospital and £6 10s. per bed at St. Thomas's and at the Homœopathic Hospital. The cost of the hospital laundry varies between £10 per bed at the Poplar Hospital and £7 per bed at the Royal Free. The larger the hospital, the smaller in proportion the laundry expenses should be, since there are many administrative expenses which, spread over a large amount of work, tend to diminish the averages. It having, then, been ascertained that a private laundry is not a costly luxury, even when built and managed on thoroughly modern lines, it remains to consider what advantages it can claim over the purely commercial undertaking, which relieves the hospital of any responsibility with regard to the soiled linen from the moment when it leaves the doors till it is returned presumably clean. The chief flaw in the commercial treatment of dirty linen is that it can give no security for the linen being efficiently cleansed. The linen technically known as "foul" ought to be subjected to a double process of purification. The first is submersion for an hour in some form of disinfectant, and this process is generally carried out on the hospital premises in establishments where there is no private laundry. But even when it has been treated thus there remains a doubt whether all germs have been destroyed. Nothing can render the foul linen perfectly safe for ordinary use but exposure to a high degree of heat. This can only be ensured in a laundry managed in the interests of the hospital, fitted with washers in which the amount of steam can be controlled so that absolute disinfection is carried out. Where there are midwifery wards, it becomes a matter of urgent importance that articles intended for use in this department should not be mixed with miscellaneous washing. Who can ensure in an ordinary laundry that these precautions will be observed? A general order may, it is true, be given to this effect, but unless there is a manageress acting in the interests solely of the hospital, and properly instructed in her duties, it will be only too probable that precautions will be regarded as fads, to be evaded whenever possible. Again, the standard of washing ought undoubtedly to be higher in a private than in a public laundry. The conditions of labour will, or should, be far higher; the service should therefore attract a higher class of workwomen, and there should be an element of permanence and of continued training, such as is altogether absent, as a rule, in the commercial undertaking. A good manageress takes a pride in working up the quality of the work, and, being in immediate touch with the matron and other authorities, the success of her work is reflected upon her. It raises the whole affair into a special department in a different category altogether to that of a business wherein some unknown proprietor is endeavouring to squeeze as much profit as possible from the labours of certain employés. The gain to the hospital of a perfected organisation in respect to the removal and return of soiled linen must not be overlooked. If the laundry is well managed, the linen can be dealt with every day, and the staff and plant can be brought down to the lowest possible dimensions. Whatever is part of the daily routine in hospital work is done in an orderly manner. The use of a private laundry (under good management) removes all flurry and disorder from the perpetual collecting and distributing of the ward supplies. They come and go at given hours, and the whole machinery is under the matron's control. By this method also the wear and tear of the ordinary laundry is largely diminished. True, washing subjects every kind of article to a great strain. But the home laundry is free from many of the abuses which creep into enterprises undertaking enormous masses of work at very low contract prices. It is free, too, from the thefts which haunt the commercial laundry, where often a very low class of woman is employed at the lowest grade of wages. In hospitals despatching relays three or four times a week to outside laundries, the "lost" articles mount up to a figure which counts for a good deal in the course of a year, and experience shows that it is seldom much good making frantic efforts to recover them. The price may be made up on demand, but in some cases where the contract price is low "losses" are not made good, there being always the saving assumption that where lists do not tally, it is the sister who is at fault.

For these and many other reasons the hospital laundry ought to be under the control of the matron, with a manageress directly accountable to her for the quality of the work performed in it. Under such conditions alone can complete efficiency be maintained in this department at the lowest possible cost.

